# Research progress of N6-methyladenosine in colorectal cancer: A review

**DOI:** 10.1097/MD.0000000000036394

**Published:** 2023-11-24

**Authors:** Yu Lin, Hongjun Shi, Lianping Wu, Linyang Ge, Zengqing Ma

**Affiliations:** a Department of Respiratory, Nanjing Gaochun People’s Hospital, Nanjing, China; b Department of Pharmacy, Nanjing Gaochun People’s Hospital, Nanjing, China.

**Keywords:** clinical application, colorectal cancer, m^6^A modification, m^6^A regulators, non-coding RNAs

## Abstract

Colorectal cancer is the third most common malignant tumor worldwide, causing serious harm to human health. Epigenetic modification, especially RNA methylation modification, plays a critical role in the occurrence and development of colorectal cancer via post-transcriptional regulation of mRNA and non-coding RNA expression. Among these, N6-methyladenosine (m^6^A) is the most common chemical modification in mammals, which plays an important role in the progress of cancer, including colorectal cancer. m^6^A is a dynamic and reversible process and is mainly regulated by m^6^A methyltransferase (“writers”), m^6^A demethylases (“erasers”), and m^6^A binding proteins (“readers”). Herein, we reviewed recent advances in the role of m^6^A modification in colorectal cancer and focused on the factors affecting m^6^A modification. Furthermore, we discussed the clinical application of m^6^A modifications for colorectal cancer diagnosis, prognosis, and treatment and provided guides in clinical practice. m^6^A modification and m^6^A regulators play significant roles in the occurrence and development of colorectal cancer by regulating the stability and translation of mRNAs, the maturation of miRNAs, and the function of lncRNAs. m^6^A regulators can play biological roles in colorectal cancer through m^6^A-dependent manner or m^6^A-independent manner. Multiplies of internal factors, including miRNAs and lncRNAs, and external factors can also regulate the m^6^A modification by completing with m^6^A regulators in a base complement manner, regulating the expression of m^6^A and mutating the m^6^A site. m^6^A regulators and m^6^A modificantion are diagnostic and prognostic markers for CRC. Therefore, m^6^A regulators and m^6^A modificantion may be potential therapeutic target for CRC in the future.

## 1. Introduction

Colorectal cancer (CRC) ranks third in morbidity and second in cancer-related mortality globally.^[1]^ For CRC patients in the early stages, the 5 years survival rate can reach 90%. Unfortunately, for most cancer patients, recurrence or metastasis is inevitable. Additionally, for CRC patients with advanced stage, the option of chemotherapy regimens is limited, and immunotherapy is only suitable for a small group of cases, resulting in a 5-year survival rate of only 14%.^[2]^ Therefore, it is necessary to further reveal the mechanism of CRC initiation and development.

It is well known that the development of CRC is a complex process, including the interaction between environmental factors and genetic factors.^[3,4]^ For example, β-catenin and cyclooxygenase 2 have been demonstrated to closely participate in the occurrence and development of CRC.^[5]^ Recently, RNA methylation modification, as an important part of post-transcriptional regulation, has become an emerging research focus in the field of epigenetic inheritance in tumors. More than 100 types of chemical modifications have been identified in RNA levels,^[6]^ including mRNA N6-methyladenosine (m^6^A), N^1^-methyladenosine,^[7]^ N^7^-methylguanosine,^[8]^ 5-methylcytosine (m^5^C), and 5-hydroxymethylcytosine, etc.^[9,10]^ Among these, m^6^A modification, namely methylated at the sixth N atom of RNA adenine base, is the most abundant and accounts for 0.1% to 0.4% of the total adenine in eukaryotic messenger RNAs (mRNAs).^[11,12]^ Moreover, the m^6^A modification not only occurs in mRNAs but also in non-coding RNAs such as microRNAs (miRNAs), long non-coding RNAs (lncRNAs), and circular RNAs (circRNAs).^[13,14]^ At the transcriptome level, the deposition of m^6^A modification prefers to locate at near stop codon, coding region (CDS), and 3′untranslated terminal region (3′UTR) with the characteristic of RRACH ([G/A/U] [G > A]m^6^AC[U > A > C]) motif.^[15]^

Recently, accumulating evidence suggests that m^6^A regulators and m^6^A modification are closely linked with human cancers, including CRC. Herein, we summarize the biological function of m^6^A modification and its influencing factors in CRC. Additionally, we discuss the clinical applications of m^6^A modification in CRC.

## 2. The role of m^6^A regulators in m^6^A modification

**Figure 1.**
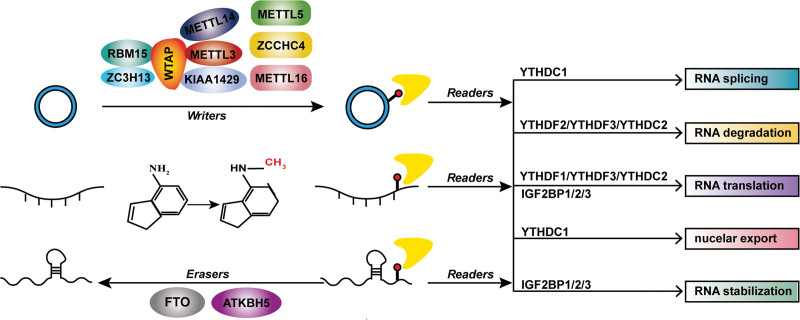
The process of m^6^A modification is regulated by “writers,” “erasers” and “readers.” The m^6^A modification is catalyzed by the m^6^A methylase complex consisting of METTL3/METTL14/WTAP/RBM15/KIAA1429 and other dependent methyltransferases such as METTL16, ZC3H13, ZCCHC4 and METTL15. The m^6^A modification can be removed by m^6^A erasers, containing FTO and ALKBH5. The fate of modified-genes is determined by m^6^A readers including IGF2 (IGF2BP1/2/3) and YTH domain (YTHDF1/2/3, YTHDC1/2), which regulates the stability, translation, export and splicing.

The process of m^6^A modification is regulated by m^6^A methyltransferase (“writers”), m^6^A demethylases (“erasers”), and m^6^A binding proteins (“readers”) (Fig. 1). Methyltransferase like 3 and 14 proteins (METTL3 and METTL14) and their cofactors Wilms tumor 1-related proteins (WTAP), KIAA1429, zinc finger CCCH domain-containing protein 13 (ZC3H13), and RNA binding motif protein 15 (RBM15) constitute the m^6^A methyltransferase complex, which catalyzes base A to form m^6^A.^[16–21]^ Recently, methyltransferase like 16 (METTL16), methyltransferase like 5 (METTL5), and zinc finger CCHC-type containing 4 (ZCCHC4) are identified as “writers” with independent catalytic activity.^[22–24]^ In contrast, fat mass and obesity-associated protein (FTO) and alkB homolog 5 (ALKBH5) remove the methyl code from targeted RNAs to maintain the balance of m^6^A modification in the host.^[25,26]^ The fates of m^6^A-modified RNA transcripts are ultimately determined by the types of m^6^A “readers” including the YT521-B homology (YTH) domain family (YTHDF1/2/3, YTHDC1/2),^[27]^ insulin-like growth factor 2 mRNA binding proteins (IGF2BP1/2/3), heterogeneous nuclear ribonucleoprotein family (HNRNPC, HNRNPG, and HNRNPCA2B1) and eukaryotic initiation factor 3 (EIF3).^[28]^ Moreover, METTL3 can also function as a reader to promote translation.^[29]^ m^6^A “readers” regulate the gene expression by recognizing and binding the m^6^A site to affect RNA splicing, degradation, stability, translation, and export.^[30]^

### 2.1. Writers

As the first identified m^6^A writer, METTL3 has the sole catalytic subunit in the complex of m^6^A “writers” and catalyzes the formation of m^6^A modification.^[17]^ METTL14 can form a stable heterodimer with METTL3 to assist in substrate recognition.^[17]^ WTPA enhances catalytic activity by recruiting the METTL3-METTL14 complex to nuclear speckle.^[19]^ RBM15 recruits the METTL3-METTL14-WTAP complex to special RNA sites by binding with them.^[20]^ KIAA1429 can mediate the complex to select m^6^A methylation in the 3′UTR or near stop codon.^[31]^ ZC3H13 promotes m^6^A modification by bridging WTAP to Nito, an mRNA-binding factor.^[21]^ Additionally, METTL16, ZCCHC4, and METTL5 possess the independent catalytic activity to catalyze m^6^A on some structured RNAs such as U6 spliceosomal RNA, 28S rRNA, and 18S rRNA, respectively.^[22–24]^

### 2.2. Erasers

FTO, the first identified m^6^A demethylase in 2011, indicates that the m^6^A modification is a dynamic and reversible process.^[25]^ However, recent studies demonstrate that FTO preferentially targets N6, 2-O-dimethyladenosine (m^6^A_m_) but not m^6^A, especially m^6^A_m_ in snRNAs.^[32]^ ALKBH5, the second identified RNA m^6^A demethylase, mainly targets m^6^A in mRNAs.^[26]^

### 2.3. Readers

YTHDF2, the first reported m^6^A reader, regulates the degradation of transcripts by bringing translatable mRNAs with m^6^A modification to mRNA decay sites and recruiting CCR4-NOT deadenylase complex to trigger deadenylation and degradation.^[27]^ In contrast, YTHDF1 recognizes m^6^A modification by binding with initiation factor eIF3 to promote the initiation of translation and protein synthesis.^[33]^ Interestingly, the function of YTHDF3 depends on the protein with which it interacts. The interaction between YTHDF3 and YTHDF2 accelerates the attenuation of m^6^A-modified mRNAs. However, YTHDF3 promotes mRNA translation when interacting with YTHDF1.^[34]^ YTHDC1 facilitates the shearing process of targeted mRNAs by recruiting the splicing factors, serine/arginine-rich splicing factor 1,3 (SRSF1 and SRSF3) and SC35.^[35]^ YTHDC1 regulates the exportation of m^6^A-modified mRNAs from the nucleus to the cytoplasm.^[36]^ YTHDC2 can promote mRNA translation and regulate mRNA degradation.

In contrast to the destabilizing function of YTHDF2/3, the insulin-like growth factor-2 mRNA-binding proteins (IGF2BPs) family, including IGF2BP1, IGF2BP2, and IGF2BP3, maintain m^6^A-modified mRNAs stability through interacting with ELAV like RNA binding protein 1 (ELAVL1, also known as HuR) or MATRIN3.^[37]^ Interestingly, cytoplasmic METTL3 can also act as a reader to promote the translation of downstream mRNAs by recruiting the translation initiation factor eIF3, which does not depend on its m^6^A methyltransferase activity.^[29]^ EIF3 could be considered as a reader of 5′UTR m^6^A to promote the initiation of translation.^[38]^

## 3. Role of m^6^A modification on mRNAs in CRC

### 3.1. m^6^A modification enhances the stability of mRNA

m^6^A modification is the most common chemical modification in mRNA.^[12]^ Numerous pieces of evidence suggest that m^6^A modification can regulate tumor-related mRNA expression by affecting RNA stability, which involves the tumorigenesis and metastasis of various tumors.

As elucidated by Shen et al METTL3 directly interacts with the 5′/3′UTR regions of hexokinase 2 and the 3′UTR region of glucose transporter 1 (GLUT1), which are respectively recognized by the m^6^A reader IGF2BP2 or IGF2BP2/3 to activate the glycolysis pathway, thus promoting CRC tumorigenesis.^[39]^ As reported by Li et al METTL3 methylates SRY-box transcription factor 2 mRNA in the coding sequence (CDS) region, which is recognized by IGF2BP2 to maintain its stability, thus promoting CRC cell stemness and driving tumorigenesis and metastasis.^[40]^ Xiang et al revealed that METTL3 functions as an oncogene in CRC by enhancing Myc expression, which accelerates cell cycle transition in an m^6^A-IGF2BP1- dependent manner.^[41]^ Zhu et al found that METTL3 promotes CRC proliferation by directly interacting with the 3′UTR of cyclin E1 (CCNE1) mRNA to enhance its stability in an m^6^A-dependent manner, which increases the CCNE1 expression and promotes cell transformation from G1 to S phase.^[42]^ The above studies consistently confirmed that METTL3 exerts an oncogene role in the occurrence and progression of CRC. However, one study found that METTL3 acts as a tumor suppressor gene in CRC proliferation and metastasis by inhibiting the activation of the p38/ERK pathway.^[43]^ The cancer heterogeneity and different downstream genes might be the reasons to explain the different roles of METTL3 in CRC. Additionally, De Filippo C et al also identified that IGF2BP2 maintains the RAF1 stability through binding to its 3′UTR, which blocks its degradation mediated by miR-195. Thus, IGF2BP2 contributes to CRC proliferation.^[44]^ Numerous studies have shown that IGF2BP3 is highly expressed in CRC tissues and is associated with adverse clinical outcomes.^[45–49]^ Recently, Xu et al first revealed the oncogenetic role of IGF2BP3 in CRC. They found that IGF2BP3 promotes the proliferation of CRC cells by regulating the cell cycle and inducing migration in an epithelial-mesenchymal transition manner. However, the underlying targeted genes have not been identified.^[50]^ Recently, Li further validated that IGF2BP3 forms a complex with RNA-binding protein ELAVL to enhance the stability of cell cycle-related genes, thereby promoting CRC proliferation.^[51]^

### 3.2. m^6^A modification reduces the stability of mRNA

Interestingly, a recent study revealed that the knockdown of METTL3 decreases the decay rate of suppressor of cytokine signaling 2 (SOCS2), leading to the up-regulation of SOCS2 protein in CRC. Therefore, METTL3 promotes the tumorigenicity of CRC via inhibiting SOCS2. This result suggested that m^6^A modification can also exert an inhibitory effect on mRNA stability.^[52]^ Consistent with this finding, the silencing of METTL14 significantly decreased m^6^A modification on SRY-box transcription factor 4 (SOX4) and increased the expression of SOX4 mRNA, which relies on the YTHDF2-dependent pathway. As a result, the down-regulation of METTL14 in CRC suppresses tumor metastasis by SOX4-modulated PI3K/AKT signal pathway and EMT process.^[53]^ Furthermore, the down-regulated ZC3H13 in CRC may have a complex role in mRNA stability. In particular, ZC3H13 suppresses CRC proliferation and invasion by decreasing the expression of Snail, Cyclin D1, and Cyclin E1 and increasing the expression of Occludin and Zo-1 to inactivate Ras–ERK signaling pathway.^[54]^ In summary, the regulation of m^6^A modification on mRNA stability might depend on the interaction between m^6^A “writers” and corresponding m^6^A “readers,” which are responsible for mRNA stability and degradation.

### 3.3. m^6^A modification promotes the translation of mRNA

Previous studies suggested that METTL3 can not only regulate the mRNA stability but also promote the mRNA translation in an m^6^A-dependent or independent manner.^[29]^ As reported by Vu et al^[55]^ in human acute myeloid leukemia cells, METTL3 promotes the translation of c-MYC, B-cell lymphoma 2 and phosphatase and tensin homolog mRNAs dependent on its methylation activity. Differently, Lin et al revealed that METTL3 in cytoplasm enhances the translation of epidermal growth factor receptor (EGFR) and TAZ by interacting with the translation initiation complex instead of the methylation activity or m^6^A regulator proteins, contributing to lung cancer cell growth and invasion.^[29]^ Chen et al found that the GLUT1 m^6^A modification mediated by METTL3 induces GLUT1 protein translation, which enhances glucose uptake and lactate production, leading to the activation of mTORC1 signaling and tumorigenesis in CRC.^[56]^ However, whether METTL3 could promote the translation of targeted genes in m^6^A independent manner in CRC is still needed to explore in the future.

## 4. Role of m^6^A modification on non-coding RNAs in CRC

### 4.1. m^6^A modification promotes the maturation of miRNAs

The miRNAs belong to non-coding single-stranded small molecular RNA with 21 to 25 nucleotides in length, which have an important role in human cancer, including proliferation, migration, invasion, and differentiation.^[57]^ They can regulate gene expression at the post-transcriptional level by binding to the 3′UTR region of the target mRNA sequence in a base-pairing manner, leading to target mRNA degradation or translational inhibition.^[58,59]^ Additionally, Meyer et al have identified a strong association between miRNA-binding sites and m^6^A-modified sites in the 3′UTR, indicating that a functional interaction may exist between m^6^A modifications and miRNAs targeted genes.^[15]^ Recently, studies have reported that m^6^A regulators can regulate miRNA biogenesis.^[60]^ In the process, miRNAs are firstly transformed into primary miRNAs (pri-miRNAs) in the nucleus, which are further processed into pre-miRNA under the assistance of a microprocessor complex containing DGCR8 and Drosha. Subsequently, pre-miRNA was transported into the cytoplasm and cleaved by Dicer into mature miRNAs.^[61]^ Intriguingly, Alarcón et al found that pri-miRNAs, methylated by METTL3, promote DGCR8 to recognize and bind the substrates with m^6^A modification instead of other secondary structures in transcripts, thus enhancing miRNA maturation.^[60]^ This revealed a novel mechanism for m^6^A regulators in human cancers. As elaborated by Peng et al METTL3 contributes to the maturation of pri-miR1246 under the assistance of DGCR8 in an m^6^A-dependent manner. The mature miR-1246 reduces the expression of an anti-oncogene sprouty related EVH1 domain containing 2, therefore interacting with the Raf/MEK/ERK pathway to promote CRC metastasis.^[62]^ METTL14 has also been demonstrated to regulate the maturation of miRNAs and identified as a tumor suppressor in CRC. Chen et al found that METTL14 positively mediates primary miR-375 maturation by DGCR8 in an m^6^A-dependent manner. The accumulated miR-375 targets Yes1 associated transcriptional regulator to inhibit proliferation ability and Sp1 transcription factor (SP1) to suppress migration and invasion ability, respectively.^[63]^

### 4.2. m^6^A modifications are indispensable for lncRNA function

LncRNAs are transcripts with a length of more than 200 nucleotides and lack protein-coding capacity.^[64]^ They can act as oncogenes or suppressors in tumorigenesis and progression via various mechanisms to modulate gene expression.^[65,66]^ Recently, lncRNAs have also been demonstrated to exist in m^6^A modification extensively.

As reported by Wu et al lncRNA RP11 is highly expressed in CRC and promotes the dissemination of CRC cells. The upregulation of RP11 in CRC can account for m^6^A modification other than DNA methyltransferase or histone acetylation, which triggering the RP11 localization to chromatin. In detail, overexpression of METTL3 increased RP11 expression, while overexpression of ALKBH5 decreased its expression. Mechanically, m^6^A-modified RP11 forms a complex with heterogeneous nuclear ribonucleoprotein A2/B1 (hnRNPA2B1) and mRNA (RP11/hnRNPA2B1/mRNA), promoting siah1 and fbxo45 degradation and subsequently blocking the mesenchymal transition-related gene zeb1degradation.^[67]^

LncRNA XIST, a novel discovered lncRNA, acts as an oncogene in various cancer, including CRC.^[68]^ Evidence suggests that m^6^A modification also exists at the special sites of lncRNA XIST. The m^6^A-modified XIST suppresses the gene transcription on the X chromosome, but the potential mechanisms are unclear.^[69]^ In detail, the knockdown of RBM15 and RBM15B or METTL3 impairs XIST-mediated gene silencing. Further functional studies show that YTHDC1, an m^6^A “reader” protein, recognizes the m^6^A site on XIST and maintains its function.^[68]^ Recently, another study revealed that METTL14 plays an anti-oncogene role in CRC by targeting the lncRNA XIST. Mechanically, m^6^A-modified XIST can be recognized and bonded by YTHDF2, which promotes XIST degradation.^[70]^ This study emphasizes the inhibitory effect of METTL14 in CRC and reveals a novel mechanism of m^6^A in CRC, which may provide a new sight for CRC therapeutic strategies in the future.

Additionally, Ni et al found that lncRNA GAS5 promotes the transport of endogenous YAP from the nucleus to the cytoplasm by binding with the WW domain of YAP. In the cytoplasm, GAS5 activates YAP phosphorylation and its ubiquitin-mediated degradation to suppress the progression of CRC. Intriguingly, m^6^A reader protein YTHDF3 is not only a novel identified target of YAP but also promotes m^6^A-modified lncRNA GAS5 degradation, which uncovers a negative feedback loop of lncRNA GAS5-YAP-YTHDH3 axis in CRC.^[71]^

### 4.3. m^6^A modification promotes cytoplasmic export of circRNAs in CRC

CircRNAs, a class of small non-coding RNAs with a covalent single-stranded circular structure, are the backing-splicing or skipping products of precursor mRNAs.^[72]^ It exists widely in various eukaryotes and involves distinct biological processes by acting as miRNA sponges.^[73]^ However, m^6^A modification shows different patterns between circRNAs and mRNAs. In general, m^6^A-circRNAs normally occur at exons that have no m^6^A modification in mRNAs, indicating that m^6^A-circRNAs occurred during or after circRNAs formation, but the potential mechanisms are needed to further explore.

Recently, Chen et al uncovered that m^6^A modification regulates the cytoplasmic export of circNSun2, a crucial oncogenic circRNA in CRC cells. In the nucleus, m^6^A-modified circNSun2 can interact with YTHDC1 at the exon5-exon4 junction site with the GAACU m^6^A motif, which promotes circNSun2 to export to the cytoplasm. Subsequently, circNSun2 enhances the stability of high mobility group AT-hook 2 (HMGA2) mRNA under the assistance of the circNSun2/IGF2BP2/HMGA2 complex, promoting liver metastasis. In addition, the up-regulation of m^6^A-modified circNSun2 has been observed in tumor tissues and serum samples of patients with CRC with liver metastasis, suggesting that circNSun2 may become a new diagnostic or prognostic biomarker and a potential therapeutic target for CRC with liver metastasis.^[74]^

Several m^6^A regulators have been demonstrated to play a crucial role in the progression of CRC. However, the corresponding downstream targeted genes are still unclear. For example, Yang et al found that the expression of ALKBH5 is downregulated in colon cancer and shows a beneficial prognostic value. Functional studies suggested that overexpression of ALKBH5 impairs the ability of colon cells to invade and metastasize,^[75]^ indicating that ALKBH5 may be a potential therapeutic target. Considering the function of ALKBH5 in CRC, it is meaningful to further explore the underlying mechanism in the future.

**Figure 2.**
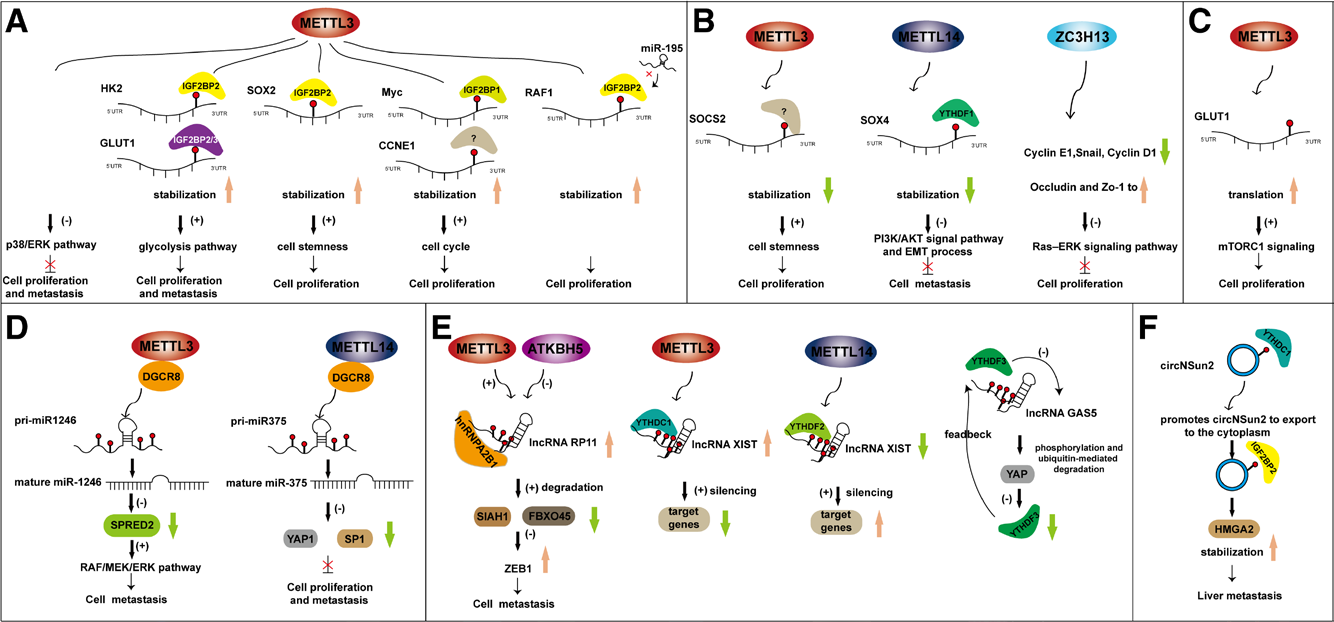
The function of m^6^A regulators on mRNAs and non-coding RNAs in colorectal cancer. The m^6^A regulators enhance (A) or reduce (B) the mRNAs stability; (C) The m^6^A regulators promote the mRNAs translation; (D) The m^6^A regulators promote the maturation of microRNAs; (E) The m^6^A modification maintains the function of lncRNAs; (F) The m^6^A modification promotes circRNAs export to cytoplasm.

As described above, the m^6^A regulators can regulate various biological functions of mRNAs and non-coding RNAs to participate in the occurrence and development of CRC (Fig. 2; Table 1).

**Table 1:** The function of m^6^A regulators in colorectal cancer.

Proteins	Targeted genes	Function	Functional description	Mechanism	Reference
METTL3	HK2, GLUT1	Oncogene	Promoting CRC tumorigenesis	Enhancing the stability of HK2 (METTL3/HK2/IGF2BP2) and GLUT1 (METTL3/GLUT1/IGF2BP2/3) which activates the glycolysis pathway	^[41]^
	SOX2	Oncogene	Promoting cell stemness and drive tumorigenesis and metastasis	Methylating SOX2 mRNA in the CDS region, which is recognized by IGF2BP2 to maintain its stability (METTL3/SOX2/IGF2BP2)	^[42]^
	Myc	Oncogene	Promoting CRC cell proliferation	Enhancing the myc expression in an m^6^A-IGF2BP1- dependent manner, which accelerates cell cycle transition (METTL3/myc/IGF2BP1)	^[43]^
	CCNE1	Oncogene	Promoting CRC cell proliferation	Methylating 3′UTR of CCNE1 mRNA to enhance its stability, which promotes cell cycle transition	^[44]^
	SOCS2	Oncogene	Promoting cell proliferation	Maintaining the tumorigenicity of colon cancer cells by suppressing SOCS2	^[54]^
	GLUT1	Oncogene	Promoting CRC tumorigenesis	Promoting GLUT1 translation, which subsequently accelerates glucose uptake and lactate production, leading to the activation of mTORC1 signaling (METTL3-GLUT1-mTORC1)	^[58]^
	P38/ERK	Tumor suppressor	Suppressing cell proliferation and metastasis	Inhibiting the activation of p38/ERK pathway	^[45]^
	miR-1246	Oncogene	Promoting cell metastasis	Promoting the maturation of miR-1246, which reduces SPRED2 expression to activate the MAPK pathway	^[64]^
	lncRNA RP11	Oncogene	Triggering the cell dissemination	m^6^A-modified RP11 forms a complex with hnRNPA2B1, promoting siah1 and fbxo45 degradation and blocking zeb1degradation (RP11/hnRNPA2B1/ siah1 and fbxo45)	^[69]^
METTL14	SOX4	Tumor suppressor	Suppressing CRC metastasis	Reducing the stability of SOX2 (METTL14/SOX4/YTHDF2), which modulates PI3K/AKT signal pathway and EMT process	^[54]^
	miR-375	Tumor suppressor	Suppressing tumor proliferation and metastasis	Promoting the maturation of miR-375, then the accumulated miR-375 targets YAP1 and SP1	^[65]^
	lncRNA XIST	Tumor suppressor	Suppressing proliferation and metastasis	m^6^A-modified XIST can be recognized and bond by YTHDF2, which promotes the oncogenic XIST degradation (METTL14/lncRNA XIST/YTHDF2)	^[72]^
ZC3H13	Snail, Cyclin D1, and Cyclin E1; Occludin and Zo-1	Tumor suppressor	Suppressing CRC proliferation and invasion	Regulating tumor-related genes, such as decreasing the expression of Snail, Cyclin D1, and Cyclin E1, and increasing the expression of Occludin and Zo-1 to inactivate Ras–ERK signaling pathway	^[55]^
FTO	Mcy	Oncogene	Promoting CRC cell proliferation and invasive	miR-96 down-regulates AMPKα2, which increases expression of FTO and subsequently upregulates expression of MYC via blocking its m^6^A modification	^[76]^
ALKBH5	Non-identified	Tumor suppressor	Suppressing cell invasion	Unclear	^[77]^
IGF2BP2	RAF1	Oncogene	Promoting CRC proliferation	Maintaining the RAF1 stability through blocking its degradation mediated by miR-195.	^[46]^
	MYC	Oncogene	Promoting CRC cell proliferation	Interacting with m^6^A-modified MYC to activate glycolysis metabolism	^[78]^
IGF2BP3	Non-identified	Oncogene	Promoting CRC cell proliferation	Regulating cell cycle and inducing migration in an EMT manner	^[52]^
	Cell cycle related genes	Oncogene	Promoting CRC cell proliferation	Forming a complex with RNA-binding protein ELAVL to enhance the stability of cell cycle-related genes	^[53]^
m^6^A modification	lncRNA GAS5	Tumor suppressor	Suppressing proliferation and metastasis	m^6^A-modified GAS5 activates YTAP phosphorylation and its ubiquitin-mediated degradation (lncRNA GAS5-YAP-YTHDH3)	^[73]^
m^6^A modification	circNSun2	Oncogene	Promoting liver metastasis	Promoting circNSun2 to export to the cytoplasm. circNSun2 enhances the stability of HMGA2 mRNA under the assistance of the circNSun2/IGF2BP2/HMGA2 complex	^[79]^
m^6^A modification	HSF1	Oncogene	Promoting CRC development	β-catenin suppressed the biogenesis of HSF1-targeting miR455-3p to favor METTL3 binding and m^6^A modification of HSF1 mRNA, thus promoting HSF1 translation	^[80]^

## 5. Factors affecting m^6^A modification or m^6^A regulators expression in CRC

**Figure 3.**
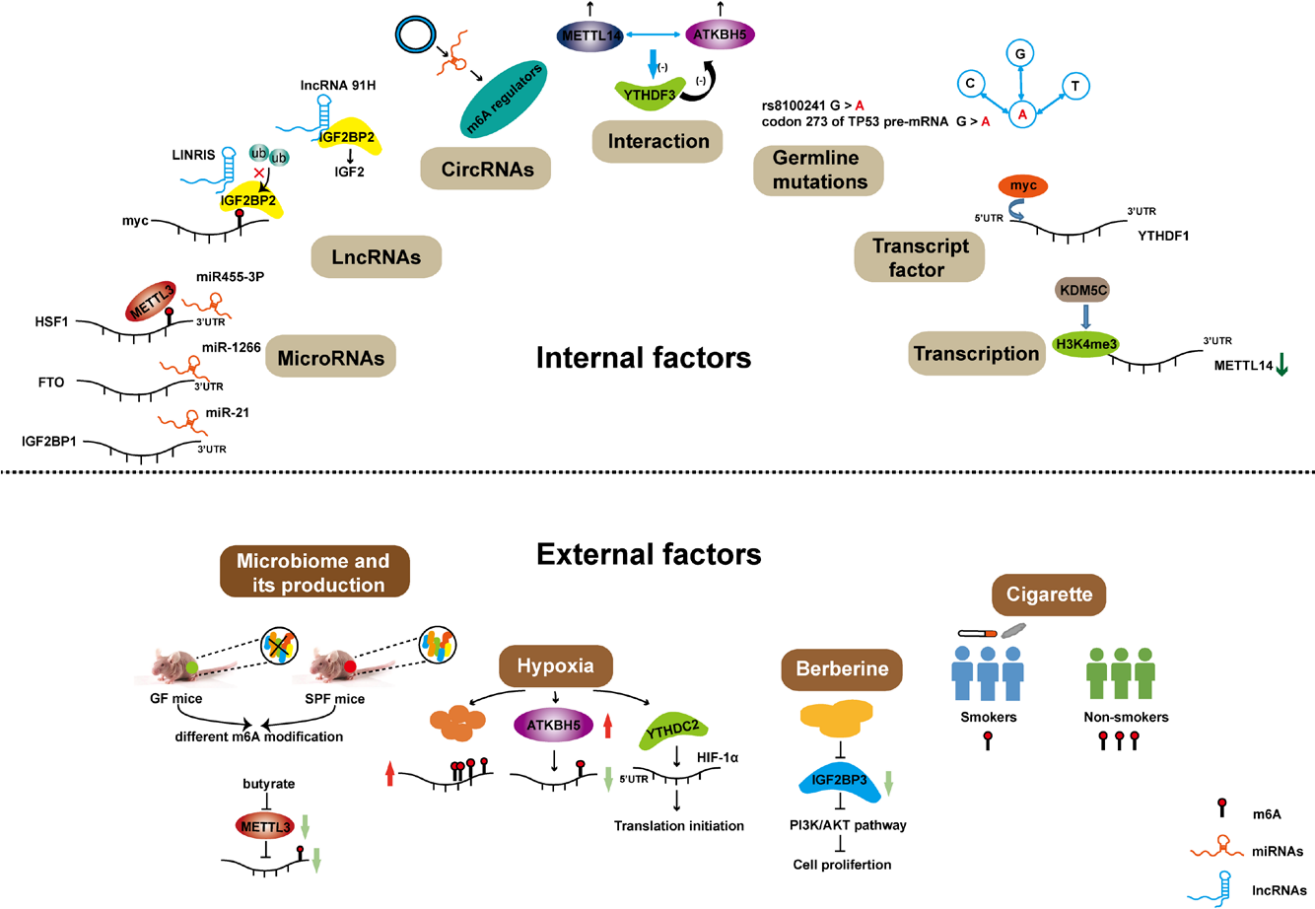
The factors regulating m^6^A modifications or m^6^A regulators expression in colorectal cancer.

Considering the important role of m^6^A modification and m^6^A regulator proteins in CRC, it is of great significance to explore the factors which can affect it. We summarize the research findings in recent years and elaborate on the possible factors from 2 aspects: internal and external factors (Fig. 3).

### 5.1. Internal factors

#### 5.1.1. miRNAs complete with m^6^A modification or regulate m^6^A regulators expression.

Evidence suggests that the majority of the m^6^A peak is located at the 3′UTR of mRNAs, where also contains the miRNAs bind sites.^[15]^ This indicates that, on the one hand, the m^6^A peak may be targeted by miRNAs in a base complement manner; on the other hand, m^6^A regulators and miRNAs may compete with each other to regulate the downstream target genes. As reported by Chen et al even under the strict alignment criteria, there are still over 90% of m^6^A peaks that could pair with miRNAs. The further functional study demonstrated that miRNAs could regulate the m^6^A methyltransferase activity of METTL3 by affecting its binding to the m^6^A site on the 3′UTR region of target mRNAs in a sequence pairing mechanism.^[79]^ Similarly, miR455-3P inhibits the translation of heat shock transcription factor 1 (HSF1) mRNA by interacting with its 3′UTR with base pairing. Intriguingly, HSF1 mRNA 3′UTR also contains an m^6^A motif that can be bound by METTL3. The overexpression of miR455-3p reduces the combination of METTL3 with HSF1 and decreases the m^6^A modification on HSF1 mRNA. Moreover, the depletion of METTL3 not only decreases the m^6^A-modified HSF1 mRNA but also promotes the interaction of miRNA455-3p with HSF1 mRNA. These findings implied that miR455-3p can complete with METTL3 in HSF1 mRNA m^6^A modification.^[77]^ Additionally, miRNAs can also directly regulate m^6^A regulators by binding with their 3′UTR. Shen et al found that miRNA-1266, with low expression in CRC, negatively regulated the FTO expression by a sequence pairing mechanism at 3′UTR, thus increasing the expression of FTO and contributing to the progression of CRC.^[81]^ However, whether miRNA-1266 regulates the methylation modification function of FTO is still unclear. Additionally, Xie et al also found that miRNA-21, as an oncogene in CRC, interacts with 3′UTR of IGF2BP1 to reduce its expression in a sequence pairing mechanism.^[80]^ Recently, Yue et al discovered that miRNAs can also indirectly affect the m^6^A regulators. In detail, miR-96 inhibits the AMPKα2, which upregulates the FTO expression. Subsequently, FTO decreases the m^6^A modification of myc to enhance its expression, thus promoting CRC cell proliferation and invasion.^[82]^ These researches suggested that in CRC, miRNAs can compete with m^6^A regulators to affect the m^6^A modification of the downstream targeted genes. Meanwhile, miRNAs can also regulate the m^6^A expression directly or indirectly.

#### 5.1.2. LncRNAs maintain m^6^A regulators expression.

The lncRNA LINRIS, a highly expressed oncogenic lncRNA in CRC, blocked the K139 ubiquitination of IGF2BP2 to hander its degradation via the autophagy-lysosome pathway, maintaining the IGF2BP2 expression in CRC. Then, IGF2BP2 interacted with m^6^A-modified myc to activate glycolysis metabolism, promoting CRC cell proliferation.^[83]^ Gao et al first found that lncRNA 91H expression is elevated in CRC, which can interact with m^6^A reader protein IGF2BP2 to upregulate insulin-like growth factor 2 (IGF2) expression, causing tumor metastasis.^[76]^

#### 5.1.3. CircRNAs indirectly regulate the expression of m^6^A regulators by targeting microRNAs.

CircRNAs, as miRNA sponges, can inhibit the interaction between miRNAs and their downstream targeted genes.^[78]^ Therefore, we speculated that circRNAs might indirectly regulate m^6^A regulators and modifications by miRNAs. Jin et al found that circHIPK3 interacts with miR-654 to increase IGF2BP3 expression, which enhances the proliferation and metastasis of glioma cells.^[84]^ Additionally, Zeng et al elucidated that circHIPK3 is also highly expressed in CRC tissues and acts as an oncogene role in CRC by targeting miR-7.^[85]^ Although the role of circRNAs on m^6^A regulators has not been reported in CRC, based on the above-mentioned research, we speculate that circRNAs may participate in the regulation of m^6^A modification via miRNAs in CRC, and this needs further experiments to validate.

#### 5.1.4. The interaction between m^6^A regulators.

As we described above, the m^6^A modification is a reversible process and is regulated by 3 enzymes (Writers, Erasers, and Readers) in a dynamic equilibrium state. Once the balance is broken, it will cause disease, including the occurrence and development of cancer. Considering the different roles of m^6^A regulators in m^6^A modification, we wonder whether m^6^A regulators can interact with each other to control the m^6^A modification. As supported by Panneerdoss et al they found that METTL14 and ALKBH5 can regulate each other’s expression in breast cancer cells to affect the m^6^A modification of target genes and inhibit m^6^A reader protein YTHDF3, which in turn blocks RNA demethylase activity. In detail, the levels of METTL14 decreased with the knockdown of ALKBH5, and the expression of ALKBH5 decreased with the silencing of METTL14. Meanwhile, the expression of ALKBH5 and METTL14 increased after the overexpression of METTL14 or ALKBH5, respectively. Furthermore, METTL14/ALKBH5 formed a complex with HuR to maintain its stability and promoted tumor progression by regulating the cell cycle and TGFβ signaling–associated transcripts.^[86]^ These findings suggest that maintaining m^6^A modification at optional levels is crucial for human health and provide a novel sight for m^6^A the writers-erasers-readers axis in the CRC.

#### 5.1.5. Germline mutations affect the m^6^A binding sites.

It is well known that m^6^A modification is usually located in the conservative sequence “RRACH.”^[15]^ Therefore, the mutations in the m^6^A sites of transcripts may disturb m^6^A deposition. The rs8100241 is 3-bp away from a predicted m^6^A site annotated by the “m^6^AVar” database. Bian found that rs8100241 G > A change can significantly increase the m^6^A level and protein expression of ankyrin repeat and LEM domain containing 1 (ANKLE1), a midbody-tethered DNA nuclease, which suppresses cell proliferation by maintaining genome integrity and reduces the risk of CRC. Further studies reveal that ANKLE1 can be methylated by the METTL3/METTL14/WTAP complex and then be recognized by YTHDF1 to regulate its transcriptional efficiency.^[87]^ In addition, the G > A change in codon 273 of tumor protein p53 pre-mRNA was also reported. This mutation regulated by METTL3 promoted splicing and led to multidrug resistance in colon cancer cells.^[88]^ Above these results suggest that the mutation creates an m^6^A site on targeted genes. However, the loss of de novo m^6^A sites due to mutations has not been reported in CRC.

Additionally, the mutation that occurred in m^6^A regulators has also been reported. Meng et al have investigated the association between genetic variants in m^6^A regulators and the risk of CRC in the Chinese population. They found that staphylococcal nuclease and tudor domain containing 1 (SND1) rs118049207 was significantly associated with an incremented risk for patients with CRC, especially in men and drinkers. Mechanically, rs118049207 A allele enhances the SND1 expression by binding with doublesex and mab-3 related transcription factor 3 on this region. They also found that SND1 preferentially binds to RNA containing m^6^A in CRC cells.^[89]^ Yamaji also found that patients with A allele in FTO rs8050136 showed a higher risk of CRC compared with individuals with T allele. Moreover, rs1421085, rs1558902, rs1121980, rs3751812, rs9941349, and rs9939609 also showed positive association with the risk of CRC.^[90]^

#### 5.1.6. Transcription factors regulate m^6^A regulators expression.

Transcription factors are a group of protein molecules that can specifically bind to a specific sequence upstream of the 5′terminal of the gene to regulate its transcription.^[91]^ Recently, the regulation of transcription factors on m^6^A regulators has been reported. YTHDF1 is highly expressed in CRC and is linked with poor survival. Functional studies suggested that YTHDF1 promotes CRC proliferation. Interestingly, they found that c-myc, an oncogenic transcription factor, can bind in the 5′UTR of YTHDF1 but not others in the YTH family to promote its transcription in CRC, implying the critical role of c-myc in YTHDF1 expression.^[92]^ However, this study did not explore whether the regulation of c-Myc in YTHDF1 influences the m^6^A modification.

#### 5.1.7. KDM5C inhibits METTL4 transcription by H3K4me3.

Lysine demethylase 5C (KDM5C) is a member of KDMs family and inhibits gene transcription by catalyzing H3K4me3 demethylation.^[93]^ As reported by Chen et al the promoter region of METTL14 was enrichment with H3K4me3. Knockdown of KDM5C remarkably elevated the H3K4me3 signals in the promoter region of METTL14 and upregulated the METTL14 expression. This result suggests that KDM5C decreases the H3K4me3 may account for the low expression of METTL14 in CRC.^[53]^

### 5.2. External factors

#### 5.2.1. Microbiome and its products.

The microbiome is closely associated with the initiation and progression of various cancer, especially in CRC.^[94]^ Recently, the interaction between intestinal flora and m^6^A modification has gradually been uncovered. Wang reported that the expression of several m^6^A regulators showed a significant difference between 2 groups, germ-free mice and pathogen-free mice, suggesting that the microbiome has a strong effect on m^6^A modification.^[95]^ However, the mechanism was still unknown. A growing number of studies have shown that human intestinal microbiota can regulate the occurrence and progression of CRC by their metabolites.^[96]^ Consistently, Zhu et al discovered that the butyrate, classical production of the microbiome, decreased m^6^A levels and METTL3 expression in CRC cells in a dose-independent manner. Rescue experiments showed that overexpression of METTL3 reversed the inhibition of CRC cell proliferation caused by butyrate, indicating the important role of metabolites in m^6^A modification.^[42]^ Previous studies reported that butyrate can act as a histone deacetylase inhibitor to epigenetically regulate gene expression.^[97]^ Therefore, we speculated that the butyrate may regulate METTL3 expression and m^6^A modification in a histone deacetylase manner. However, the underlying mechanisms are also needed to explore in the future.

#### 5.2.2. Hypoxia.

Response to hypoxia is an important metabolic process in human cancers, which promotes tumor migration by regulating metastasis-related pathways such as the degradation of the extracellular matrix and the formation of tumor angiogenesis.^[98,99]^ Recently, studies showed that m^6^A undergoes drastic reprogramming under a hypoxic response. Fry et al reported that hypoxia increases the m^6^A levels of mRNAs, which subsequently enhances the stability of mRNA and promotes the recovery of translation efficiency.^[100]^ However, Wang et al found that the m^6^A levels of mRNAs were downregulated, which may be induced by ALKBH5 under cellular hypoxia conditions. Furthermore, the expression of m^6^A readers remarkably decreased, mainly including YTH family members.^[101]^ m^6^A reader YTHDC2 is also identified as a member of the DExD/H-box family of ATP-dependent RNA Helicase. Sahara, Hiroeki and his workers uncovered that YTHDC2 could unwind the 5′UTR of hypoxia inducible factor 1 subunit alpha and twist family bHLH transcription factor 1 to promote translation initiation under hypoxia condition, thus contributing to metastasis of CRC.^[102]^

#### 5.2.3. Berberine.

Berberine, an isoquinoline alkaloid, is the basic component of Coptidis Rhizoma with multiple pharmacological activities.^[103]^ Zhang et al found that berberine decreases the IGF2BP3 expression and inhibits the activity of the PI3K/AKT pathway in CRC, leading to the block of cycle transition and inhibitor of proliferation.^[104]^

#### 5.2.4. Cigarette.

Furthermore, cigarettes have also been demonstrated to show the effect on m^6^A modification. Kupsco et al found that the total RNA m^6^A levels were lower in men with long-term smoking by detecting the levels from the peripheral blood samples.^[105]^ Zhang et al found that cigarette induces METTL3 hypomethylation, resulting in the upregulated expression of METTL3, which promotes oncogenic miR-25-3p maturation and contributes to the progression of pancreatic cancer.^[106]^ However, the association between cigarettes and m^6^A modification in CRC is needed to explore.

In summary, many factors have been demonstrated to regulate m^6^A modification and regulators in CRC by multiple mechanisms. More factors and the interaction between internal and external factors are needed to be explored in the future, which will be beneficial for us to understand the function mechanism of m^6^A in CRC.

## 6. Potential clinical application of m^6^A in CRC

### 6.1. m^6^A as diagnostic and prognostic markers for CRC

m^6^A modification or expression of m^6^A regulators is usually dysregulated in CRC, and numerous pieces of evidence have suggested that m^6^A is closely associated with the initiation and progression of CRC. Thus, it seems that m^6^A might be a valuable diagnostic or prognostic biomarker.

Circulating tumor cells (CTCs) are a class of tumor cells that exist in human peripheral blood, reflecting the progression of cancer and treatment effect to some extent.^[107]^ Notably, CTCs are relatively stable structures and promising noninvasive biomarkers. Huang et al found that m^6^A levels in CTCs are significantly upregulated compared with that in whole blood cells, indicating that early detection of m^6^A levels in CTCs is of great significance for predicting prognosis and disease progression.^[108]^ Consistently, a recent study uncovered that the level of m^6^A-modified cicNsun2 was frequently increased in serum samples for CRC patients with liver metastasis. Besides, patients with high levels of m^6^A-modified cicNsun2 show poor survival.^[74]^ These findings provide a novel biomarker for monitoring the occurrence and development of CRC.

Additionally, bioinformatics analysis conducted by Liu et al revealed that the major m^6^A regulators dramatically increased in colorectal tumor tissues compared with the normal tissues. While METTL14, ALKBH5, and YTHDF3 significantly decreased in CRC tissues. The expression of WTAP, METTL16, YTHDC1, and HNRNPC is upregulated in colon cancer but not in rectum cancer. However, no obvious difference in FTO was observed between CRC and normal tissues. Moreover, they also found that the expression of m^6^A regulators is associated with the clinical characteristics and survival of CRC patients.^[109]^ So far, the expression pattern and prognostic values of several m^6^A regulators have been validated in CRC clinical samples. METTL3 was highly expressed in CRC tissues and associated with poor survival.^[40,42,62]^ The higher expression of METTL14 shows a negative association with poor overall survival in CRC patients.^[63,70]^ The upregulation of YTHDF3, YTHDF1, IGF2BP2 or the downregulation of ALKBH5 is significantly related to poor overall survival in CRC patients.^[71,75,83,92,110]^ Collectively, m^6^A might be a potential biomarker for the diagnosis and prognosis of CRC.

### 6.2. m^6^A as a potential therapeutic target for CRC

Recently, studies have revealed the regulatory role of m^6^A in chemotherapy, targeted therapy, and immunotherapy for CRC.

Cancer stem cells possess the characteristics of self-renewal and amplification, which reduces chemosensitivity and ultimately leads to cancer recurrence.^[111]^ Studies have reported that m^6^A can regulate chemotherapy resistance by maintaining the activity of cancer stem cells in CRC. In chemotherapy-resistant tissues of colon cancer, m^6^A-modified chromobox 8 (CBX8) expression is remarkably upregulated. Further study showed that m^6^A-modified CBX8, caused by METTL3, maintains the stemness properties of colon cells and inhibits the chemosensitivity of colon cancer cells to oxaliplatin and irinotecan by upregulating leucine rich repeat containing G protein-coupled receptor 5.^[112]^ Additionally, the knockdown of YTHDF1 enhances the sensitization of CRC cells to 5-FU and L-OHP. The underlying mechanism may be the classical YTHDF1 pathway which promotes m^6^A-modified genes translation and additional studies are needed to explore this mechanism.^[92]^

Kirsten rat sarcoma 2 viral oncogene homolog (KRAS) mutation frequently occurs in CRC patients, which shows a guiding significance for anti-EGFR targeted therapy such as cetuximab and panitumumab.^[113]^ A study suggested that IGF2BP2 can bind with KRAS to regulate its expression.^[114]^ This result indicates that IGF2BP2 may involve in anti-EGFR targeted therapy in CRC.

As revealed by Han, YTHDF1 can regulate the persistent neoantigen-specific immunity in an m^6^A-dependent manner. The MC38, a mouse colon adenocarcinoma cell line, was used to construct a mouse tumor model. Compared with the wide-type mice, the antigen-specific CD8^+^ cell antitumor response was enhanced in YTHDF1 deficient mice. Furthermore, the deficiency of YTHDF1 enhanced the cross-expression of tumor antigens and the cross-activation of CD8^+^ T cells in classical dendritic cells.^[115]^ Mechanistically, the m^6^A-modified transcripts encoding lysosomal proteases can be recognized and combined by the YTHDF1. Subsequently, this transcript promoted the translation of lysosomal cathepsins in dendritic cells. They also found that the therapeutic efficacy of PD-L1 checkpoint blockade was enhanced in YTHDF1−/− mice group. This result suggested that YTHDF1 may play an important role in immunity regulation.^[115]^ Wang et al reported that inhibition of m^6^A levels, caused by the depletion of METTL3 and METTL14, promotes the sensitivity of CRC to PD-1 treatment by increasing cytotoxic tumor infiltrating CD8^+^ T cells and the production of cytokine including IFN-γ, Cxcl9, and Cxcl10. Mechanistically, the depletion of METTL3 or METTL14 enhances the stability of Stat1 and Irf1 mRNA meditating by YTHDF2, resulting in the activity of IFN-γ-Stat1-Irf1 signaling. Furthermore, they also observed that METTL3 or METTL14 show a negative correlation with STAT1 in pMMR-MSI-L CRC tumors. These findings provide insight into the application of m^6^A modification in anticancer immunotherapy for pMMR-MSI-L CRC patients.^[116]^ Intriguingly, Tsuruta et al reported that FTO upregulates the expression of PD-L1 in m^6^A-dependent manner but not the classical IFN-γ signaling pathway in colon cancer cells, which reveals a new mechanism for the regulation of FTO on PD-L1 expression.^[117]^ Several proteins are only expressed in the placenta or testis and have been identified as cancer testis-proteins, which show promising as vaccine targets.^[118]^ The m^6^A reader IGF2BP3 plays an essential role during embryogenesis. In adult human tissues, the IGF2BP3 is only expressed in the testis but not in other tissues. However, IGF2BP3 is overexpressed in the majority of CRC tissues, suggesting that IGF2BP3 may be a vaccine target.^[45]^

## 7. Conclusions and perspectives

The m^6^A modification is a reversible process and is regulated by 3 enzymes (Writers, Erasers and Readers) in a dynamic equilibrium state. Once the balance is broken, it will cause disease including the occurrence and development of cancer. In this review, we summarized the role of m^6^A modification and m^6^A regulators in CRC, factors affecting m^6^A modification and clinical application of m^6^A modification. During the past few decades, numerous studies have demonstrated that m^6^A modification and its regulators play significant roles in the occurrence and development of CRC through various mechanisms. In addition to mRNAs, non-coding RNAs can all be regulated by m^6^A methylation at the post-transcriptional level. The m^6^A modification can regulate the stability and translation of mRNAs, the maturation of miRNAs, and the function of lncRNAs. Most studies have revealed that m^6^A regulators play biological roles in CRC in an m^6^A-dependent manner. However, Ma et al^[119]^ reported that KIAA1429 could also act as an RNA-binding protein to promote CRC proliferation by reducing the WEE1 mRNA stability in an m^6^A independent manner. They found that the m^6^A modification of WEE1 mRNA can be regulated by METTL3 but not KIAA1429. Furthermore, the amount of WEE1 mRNA bound to KIAA1429 did not change significantly after METTL3 knockdown. This indicated that m^6^A regulators may also participate in CRC by playing a role expect regulating m^6^A modification. Multiplies of internal factors, including miRNAs and lncRNAs, can regulate the m^6^A modifications by different mechanisms, including completing with m^6^A regulators in a base complement manner, regulating the expression of m^6^A and mutating the m^6^A site. However, related studies are rarely in CRC. Further studies are needed to investigate the m^6^A modification and m^6^A regulators in CRC development, as well as its affected factors, which will benefit for us in understanding the significance of m^6^A in the CRC process.

The dysregulation of m^6^A modification and m^6^A regulators are associated with the diagnosis and prognosis of CRC. These related m^6^A regulators might be potential therapeutic targets. More efforts are required to develop specific inhibitors targeting m^6^A regulators for clinical application in the future.

## Author contributions

**Conceptualization:** Yu Lin, Zengqing MaFunding acquisition: Linyang Ge, Zengqing Ma.

**Supervision:** Yu Lin.

**Validation:** Linyang Ge.

**Visualization:** Lianping Wu.

**Writing – original draft:** Yu Lin.

**Writing – review & editing:** Hongjun Shi, Zengqing Ma.
